# Polypharmacology or Promiscuity? Structural Interactions of Resveratrol With Its Bandwagon of Targets

**DOI:** 10.3389/fphar.2018.01201

**Published:** 2018-10-24

**Authors:** Uzma Saqib, Tanya T. Kelley, Siva K. Panguluri, Dongfang Liu, Rajkumar Savai, Mirza S. Baig, Stephan C. Schürer

**Affiliations:** ^1^Discipline of Chemistry, Indian Institute of Technology Indore, Indore, India; ^2^Department of Molecular and Cellular Pharmacology, University of Miami Miller School of Medicine, Miami, FL, United States; ^3^Department of Pharmaceutical Science, University of South Florida, Tampa, FL, United States; ^4^Center for Inflammation and Epigenetics, Houston Methodist Research Institute, Houston, TX, United States; ^5^German Center for Lung Research (DZL), Department of Lung Development and Remodeling, Max Planck Institute for Heart and Lung Research, Bad Nauheim, Germany; ^6^Discipline of Bioscience and Biomedical Engineering, Indian Institute of Technology Indore, Indore, India; ^7^Center for Computational Science, University of Miami, Coral Gables, FL, United States

**Keywords:** resveratrol, polypharmacology, protein targets, receptor, repurposing

## Abstract

Resveratrol (3, 4′, 5-trihydroxy-trans-stilbene) is a natural phytoalexin found in grapes and has long been thought to be the answer to the “French Paradox.” There is no shortage of preclinical and clinical studies investigating the broad therapeutic activity of resveratrol. However, in spite of many comprehensive reviews published on the bioactivity of resveratrol, there has yet to be a report focused on the variety and complexity of its structural binding properties, and its multi-targeted role. An improved understanding of disease mechanisms at the systems level has enabled targeted polypharmacology to mature into a rational drug discovery approach. Unlike traditional hit-to-lead campaigns that typically optimize activity and selectivity for a single target, polypharmacological drugs aim to selectively target multiple proteins, while avoiding critical off target interactions. This strategy bears promise of improved efficacy and reduced clinical attrition. This review seeks to investigate whether the bioactivity of resveratrol is due to a polypharmacological effect or promiscuity of the phenolic small molecule by examining the modes of binding with its diverse collection of protein targets. We focused on annotated targets, identified via the ChEMBL database, and matched these targets to a representative structure deposited in the Protein Data Bank (PDB), as crystal structures are most informative in understanding modes of binding at the atomic level. We discuss the structural aspects of resveratrol itself that permits binding to multiple proteins in various signaling pathways. Furthermore, we suggest that resveratrol’s bioactivity is a result of scaffold promiscuity rather than polypharmacology, and the variety of binding modes across targets display little similarity in the pattern of target interaction.

## Introduction

**FIGURE 1:**
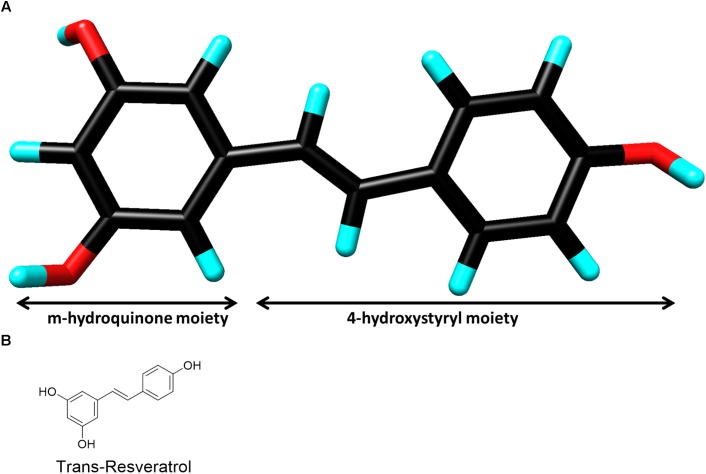
**(A)** Three-dimensional chemical structure of resveratrol. Carbon (black), oxygen (red) and hydrogen (cyan). The two chemical moieties are shown as marked. (Image created by Discovery Studio Visualizer 4.5, Accelrys). **(B)** Two-dimensional chemical structure of resveratrol.

Resveratrol (3, 4′, 5-trihydroxy-trans-stilbene) (Figure 1) was first isolated from the roots of *Veratrum grandiflorum* in 1940 (Takaoka, 1939), but it is renowned for being present in micromolar concentrations in red wine grape varietals (Siemann and Creasy, 1992). In fact, resveratrol is often attributed as the solution to the “French Paradox” which accounts for the lower incidence of heart disease even in cases of high saturated fat diets (Renaud and de Lorgeril, 1992). Resveratrol belongs to a class of antimicrobial compounds known as phytoalexins, which are often secondary metabolites synthesized by plants in response to sudden stress for protection, but not necessary for survival (Ren and Lien, 1997). Resveratrol has demonstrated activity in a myriad of disease models due to its ability to modulate numerous signaling pathways. This includes vasodilation (Gordish and Beierwaltes, 2014), platelet aggregation (Wang et al., 2002), cardio-protection (Wu et al., 2011), chemoprevention (Aziz et al., 2003), anti-viral (Campagna and Rivas, 2010), neuroprotection against stress and degeneration (Albani et al., 2010), anti-oxidant (de la Lastra and Villegas, 2007), and anti-inflammatory properties (Bereswill et al., 2010). These diverse beneficial effects of resveratrol are mainly driven by modulation of important proteins such as survivin (Hayashibara et al., 2002), Bcl2 (Low et al., 2010), p53 (Kai et al., 2010) sirtuins (SIRT1,3 and 5) (Bagul et al., 2015) and transcriptional factors like NF-κB (Yin and Cheng, 2005).

While evaluation of the mechanism and therapeutic potential of resveratrol continues to increase, no concrete structural basis of resveratrol’s polypharmacology has been proposed thus far. In the current review, we address the structural details of resveratrol and its binding with a diverse array of targets, which are often unrelated by sequence, structure fold, and even the local binding site and binding interactions. On the surface, the structural data points to a flexible mode of binding, suggesting that certain local structural complementarity triggers resveratrol-target interactions, suggesting there is some specificity required for binding. However, upon further investigation of protein-ligand interactions, it becomes apparent that resveratrol’s binding modes vary substantially. We focus our efforts on published resveratrol co-crystal structure targets to analyze the molecular interactions underlying resveratrol binding in an attempt to demonstrate its interfamily target promiscuity through non-conserved modes of binding.

## Methods for Target Selection

Resveratrol bioactivity has been widely explored. To obtain an overview of resveratrol’s reported known targets, we queried the ChEMBL 21 database and extracted all existing records as of 2018 (Gaulton et al., 2017). These were filtered by organism: *Homo sapiens*; reported assay result/endpoint: IC50, EC50, AC50, Ki, Kd; potency, unit: nM; and only records with exact endpoint values (relation: =) and confidence score ≥ 5 were kept. As we are interested in specific targets of resveratrol, we filtered and aggregated all the data records in ChEMBL 21 to include biochemical data for specific single protein targets. All records were aggregated (as arithmetic average) based on unique protein target and reported endpoint. The *p*-values are summarized in Supplementary Table 1 by protein as indicated by name and symbol. These targets were then matched with a representative structure deposited in the PDB. Table 1 describes the targets discussed in this review, along with function of resveratrol on these targets, UniProt protein names and gene names. All targets were prepared using the Schrodinger Maestro suite 2017 Protein Preparation Wizard to determine optimal protonation states of residues and ligands, as well as establishing correct bond order to HET groups, and performing a restrained minimization^1^.

**Table 1:** Various classes of resveratrol targets and their function discussed in the review.

No	Synonym	Protein name	UniProt ID	PDB ID	Class	Function
1	LTA4H	Leukotriene A4 hydrolase	P09960	3FTS	Hydrolase	Inhibitor
2	PLA2	Phospholipase A2	D0VX11	4QER	Hydrolase	Inhibitor
3	F1-ATPASE	ATP synthase subunit alpha	P19483	2JIZ	Hydrolase	Inhibitor
4	SULT1B1	Sulfotransferase family cytosolic 1B member 1	O43704	3CKL	Transferase	Inhibitor
5	QR2	NRH dehydrogenase [quinone] 2	P16083	1SG0	Oxidoreductase	Inhibitor
6	PPAR-γ	Peroxisome proliferator-activated receptor gamma	P37231	4JAZ	Transcription Regulation	Inhibitor
7	TTR	Transthyretin	P02766	5CR1	Transport protein	Inhibitor
8	cTnC	Troponin C	P63316	2L98	Contractile protein	Inhibitor
9	Myosin-2 motor domain	Myosin-2 heavy chain	P08799	3MNQ	Motor protein	Inhibitor
10	MAT2B	Methionine Adenosyltransferase Subunit beta	Q9NZL9	2YDX	Oxidoreductase	Inhibitor
11	Sirt5	NAD-dependent protein deacetylase sirtuin-5	Q9NXA8	4HDA	Hydrolase	Activator
12	Sirt1	NAD-dependent protein deacetylase sirtuin-1	Q96EB6	5BTR	Hydrolase	Activator
13	ERα	Estrogen receptor	P03372	4PP6	Protein binding	Modulator
14	TyrRS	Tyrosine–tRNA ligase	P54577	4Q93	Ligase	Modulator

## Resveratrol Inhibitory Binding Modes

### Leukotriene A4 Hydrolase (PDB: 3FTS)

Leukotrienes (LTs) are potent proinflammatory lipid mediators implicated in the pathogenesis and progression of atherosclerosis (Subbarao et al., 2004). Leukotriene A4 hydrolase (LTA4H) is a 69 kDa zinc-containing bifunctional enzyme with aminopeptidase and epoxide hydrolase activities. It converts the oxirane ring of LTA4 to leukotriene B4 (LTB4) (Samuelsson, 1983). LTB4 is a chemoattractant and an activator of inflammatory responses mediated by binding to G-protein-coupled receptors. Resveratrol inhibits LTA4H by binding to LTB4 binding site and thus acts as an anti-inflammatory agent (Davies et al., 2009).

The structure of resveratrol as an inhibitor with LTA4H suggests that it buries itself in the 17A° long L-shaped leukotriene A4 substrate binding cleft, where solvent accessibility is very low. It forms numerous important hydrogen bonds (H-bonds) with the binding site residues. The *m*-hydroquinone moiety of resveratrol binds to the cleft residues like Asp312, Phe362 and Val367. On the other hand, the 4-hydroxystyryl moiety forms only two important H-bond interactions with Asp375 and Gln136. Resveratrol also makes hydrophobic interactions with Gln136, Ala137, and Phe314 *via* its 4-hydroxystyryl ring and with Val367, Leu369, and Phe314 with the *m*-hydroquinone ring. It was further observed that all these critical residues involved in resveratrol binding belong to either a loop or a beta sheet.

### Phospholipase A2 (PDB: 4QER)

Phospholipase A2 (PLA2) is a superfamily of lipolytic enzymes that catalyze the hydrolysis of membrane phospholipids resulting in the release of arachidonic acid and lysophospholipids (Dennis, 1994). These compounds act like hormones in a number of physiological processes at extremely low concentrations (Murakami et al., 1999). However, at high concentrations they serve as precursors for inflammatory mediators such as eicosanoids or platelet-activating factor (PAF), hence aiding in inflammation. Resveratrol binds to PLA2 in the substrate binding cleft hence inhibiting eicosanoid production, exhibiting an anti-inflammatory activity (Shukla et al., 2015).

Analysis of the structure of the complex of PLA2 with resveratrol clearly shows that while most of the compound is substantially buried in the PLA2 site, parts of the scaffold remain surface exposed. The less polar 4-hydroxystyryl moiety is encapsulated in a buried environment in the substrate binding cleft, while the more electronegative *m*-hydroquinone ring is toward the surface of the receptor. Resveratrol formed three H-bonds, one each with Asp49, His48 and Cys45 via its 4-hydroxystyryl moiety, while the *m*-hydroquinone moiety makes a very strong H-bond with Leu2 close-by and a weak H-bond with Gly6 in the vicinity. Two important hydrophobic interactions are formed with Ile19 and Phe5 located in the vicinity, along with numerous Van der Waals interactions. All residues involved in binding to resveratrol belong to either a loop or an alpha helix (Shukla et al., 2015).

### F_1_-ATPase (PDB: 2JIZ)

The ATP synthase is a multi-subunit assembly found prominently in the inner mitochondrial membrane (Pedersen et al., 2000). Resveratrol inhibits ATP synthesis by binding to the ATP synthase (F1Fo-ATPase) found in mitochondria, along with inhibiting ATP hydrolysis by binding with its separate F1 catalytic domain (Zheng and Ramirez, 2000). During cardiac ischemia, the cardio-protective benefits of resveratrol result from inhibition of hydrolytic activity of F1Fo-ATPase (but not ATP synthesis) in mitochondria, thus preventing the destruction of ATP that leads to tissue damage (Zheng and Ramirez, 2000; Hong and Pedersen, 2008).

Resveratrol binds to a highly hydrophobic pocket in F_1_-ATPase (Gledhill et al., 2007), involving many buried residues where it acquires a slightly distorted planar conformation due to many hydrophobic interactions and H-bonds. The hydrophobic interactions occurring between the resveratrol and F_1_-ATPase involve residues Lys-260, Ile-263, TP-Val279, TP-Ala278, Glu264 *via* the *m*-hydroquinone moiety. While the hydrophobic interactions with Arg291, Ala256 and Thr259 are *via* the 4-hydroxystyryl moiety. Val-279 and TP-Glu-292 form H-bonds with the *m*-hydroquinone and 4-hydroxystyryl moiety of resveratrol, respectively (Gledhill et al., 2007). Here again, resveratrol is deeply buried inside the receptor cavity. Interestingly, all residues involved in binding resveratrol belong to an alpha helix.

### Sulfotransfase1B1 (PDB: 3CKL)

The human cytosolic sulfotransferases (hSULTs) comprise a family of 12 phase II enzymes involved in the metabolism of drugs and hormones (Blanchard et al., 2004). Sulfotransfase1B1 (SULT1B1) was originally identified as a thyroid hormone SULT. However, it was later discovered that it conjugates with phenols (e.g., 4-nitrophenol, 1-naphthol) and is very effective in the activation and modification of polycyclic hydrocarbons leading to the activation of carcinogens (Pang, 2011).

The SULT1B1-3′-phosphoadenosine 5′-phosphate(PAP)-resveratrol ternary complex structure has an Arg90 which acts a gating residue guarding the open ligand-bound and closed unbound conformations of the receptor (Pan et al., 2008, *unpublished data*). Resveratrol was found to be sandwiched in the hydrophobic binding pocket where the *m*-hydroquinone ring forms H-bonds with His109 and Thr21, while also forming pi-staking hydrophobic interactions with Phe24, Phe143, Tyr170, and Phe143. On the other hand, the 4-hydroxystyryl moiety is again tied up in hydrophobic interactions with Leu86, Leu149, Leu244 and Val248 with no neighboring residues to form any H-bonds. All residues involved in binding resveratrol belong to either an alpha helix or a loop (Pan et al., 2008, unpublished data).

### Quinone Reductase 2 (PDB: 1SG0)

Quinone reductase 2 (QR2) belongs to the mammalian quinone reductase family of enzymes responsible for performing flavin adenine dinucleotide (FAD) mediated reductions of quinone substrates (Jaiswal, 1994). QR2 is responsible for the generation of quinone free-radicals that are believed to cause several neurodegenerative diseases and helicobacter pylori infections (Wang and Maier, 2004; Wang et al., 2008). Specifically, their role in the production of oxidative stress has been discussed at length (Wang and Maier, 2004). Resveratrol binds to QR2, inhibiting quinone toxicity and has demonstrated impressive reduction of proliferation rates on a variety of cancer cell lines (Buryanovskyy et al., 2004). The chemopreventive and cardioprotective properties attributed to resveratrol are possibly the result of QR2 inhibition.

In the QR2 active site, resveratrol fits into a deep hydrophobic cleft (Buryanovskyy et al., 2004). Furthermore, it occupies a parallel conformation to the isoalloxazine ring of the bound cofactor FAD. Resveratrol adopts a perfectly flat conformation by burrowing itself at the QR2 dimeric interface. The two aryl rings interact with many neighboring residues through hydrophobic and van der Waals interactions. The *m-*hydroquinone ring forms H-bonds with Tyr132 and Asn161, while also participating in a close pi-pi stacking with Phe178 and similar hydrophobic interactions with Phe106 and Trp105. The 4-hydroxystyryl moiety binds with Tyr104 in a pi-pi stacking interaction and also with Trp105 and Phe126 through hydrophobic interactions. The 4-hydroxystyryl moiety further interacts through hydrogen bonding with Thr71 and Asp117. All receptor residues involved in binding belong to either an alpha helix or a loop.

### PPAR-γ (PDB: 4JAZ)

Peroxisome proliferator-activated receptors (PPARs) are members of the nuclear receptor family of ligand-dependent transcription factors. Three isoforms of PPAR have been identified: PPAR-α, PPAR-δ, and PPAR-γ, of which PPAR-γ is the most extensively studied subtype (Michalik et al., 2006). PPAR-γ is adipogenic and the inhibitory effect of resveratrol on adipogenesis is believed to occur through down-regulation of PPAR-γ mRNA expression in human visceral adipocytes (Fajas et al., 2001; Floyd et al., 2008). Resveratrol directly binds to and inhibits the Ligand Binding Domain (LBD) of PPAR-γ, where it binds to a deep surface cavity in one of the monomer units of the dimeric structure and not at the interface (Calleri et al., 2014). It makes several interactions via its m-hydroquinone ring including an H-bond with Ser342 and two hydrophobic interactions with Arg288 and Ile341. In order to accommodate the resveratrol rings, there is partial displacement of a few residues in the PPAR-γ LBD. The 4-hydroxystyryl ring forms a strong H-bond with Arg280 and a few hydrophobic interactions including those with Phe264, His266 and Ile281. All residues involved in binding resveratrol belong to either an alpha helix or a loop.

### Transthyretin (PDB: 5CR1)

Thyroid hormone transport protein transthyretin (TTR) is an amyloidogenic protein whose mutation or deletion is responsible for familial amyloidotic polyneuropathy (FAP) (Ando et al., 2005). The amyloidogenic potential of TTR is enhanced by a number of specific point mutations. Inhibitory activity of TTR fibrillogenesis is known for several classes of compounds, including resveratrol (Santos et al., 2016). Resveratrol binds to TTR on a shallow surface cavity unlike any of the other receptors described above (Florio et al., 2015). The few, but major interactions involved *via* the *m*-hydroquinone ring include a hydrophobic interaction with Leu110 and a hydrogen bond with Ser117. The 4-hydroxystyryl moiety binds TTR *via* Lys15 and Ala108 through hydrophobic interactions solely, with no hydrogen bonding. Interestingly, all interacting residue belong to the beta sheet of TTR binding site.

### Cardiac Regulatory Protein Troponin C (PDB: 2L98)

Cardiac troponin C (cTnC) is a Ca^2+^ signaling protein that triggers heart muscle contraction (Ebashi et al., 1967). Intracellular Ca^2+^ modulation carries risks associated with calcium overload such as cardiac arrhythmias, cell injury, or cell death. The role of cTnC as a target protein for the calcium sensitization is established (Haikala et al., 1995). Resveratrol increases the myofilament Ca^2+^-sensitivity of myocytes in guinea-pigs by modulating the cTnC -troponin I interaction through competitive inhibition to cTnC (Liew et al., 2005). Resveratrol binds to a broad and deep surface cavity of the cCTnC, where the m-hydroquinone ring is somewhat exposed to the solvent (Pineda-Sanabria et al., 2011). It undergoes a minor conformational change upon binding to the hydrophobic pocket of cCTnC. The resveratrol-cCTnC interaction site is populated primarily by hydrophobic interactions, such as interactions with side chains of Leu100, Leu121 via its m-hydroquinone ring. The 4-hydroxystyryl moiety forms a tight pi-pi stacking interaction with Phe156 and several hydrophobic interactions with Leu136, Leu117, Met157, Val160 and Phe153. Interestingly, there is no hydrogen bonding present between resveratrol and the binding site residues of cCTnC. The binding residues of cCTnC are all from the nearby alpha helix.

### Myosin-2 Motor Domain (PDB: 3MNQ)

Myosin-2 is an ATP-driven molecular motor that plays a crucial role in cell movement (Wilson et al., 2010). Besides serving their primary role in muscle contraction, they are also involved in other cellular processes such as cytokinesis, cortical tension maintenance, and neurite outgrowth and retraction. They are implicated in several human diseases such as hypertrophic cardiomyopathy, cancer, deafness and many neurological disorders (Aguilar-Cuenca et al., 2014). Movement by myosin motors is generated by the energy released from the hydrolysis of ATP by the actin-activated Mg^2+^ ATPase in the motor domain. Resveratrol binds to the myosin-ADP-P_i_ complex with high affinity and interfering with the phosphate release process.

Resveratrol binds to a shallow groove on the myosin-2 motor domain surface. The binding of to the receptor includes mainly H-bond interactions with Thr231, Lys229 and Thr274 *via* the *m*-hydroquinone ring. There are hardly any hydrophobic interactions of the *m*-hydroquinone ring with non-polar residues due to sparsity of these residues in the vicinity. However, the 4-hydroxystyryl ring does make a few weak hydrophobic interactions with the side chains of Gln662, Leu663, Lys661, and Asn234. Overall, resveratrol is bound by a small number of interactions in the myosin-2 motor domain. Uniquely, all these interacting residues are part of loops surrounding the binding area and no sole residue belongs to a structured entity like an alpha helix or a beta sheet.

### *S*-adenosylmethionine Synthetase 2 (PDB: 2YDX)

Methionine adenosyltransferase (MAT) is a key enzyme in cellular metabolism. It catalyzes the transfer of the adenosyl moiety from ATP to L-methionine to form *S*-adenosylmethionine (SAM) (Mudd and Mann, 1963). SAM plays a critical role in cellular metabolism as it is the major methyl donor for various biomolecules (proteins, DNA, RNA, phospholipids) in the cell. Its enzymatic activity is regulated by the associated subunit MAT2B (LeGros et al., 2001).

Interestingly, the structure of MAT2B with resveratrol reveals that there are non-canonical binding sites for resveratrol in MAT2B structure exhibiting different binding modes in the protein (Shafqat et al., 2013). One being in the substrate binding pocket of MAT2B (res1 site) and the other at the dimer interface (res2 site), which mimics possible contact regions for the MAT2A and MAT2B interactions.

Res1 binds in a broad but shallow pocket inside the substrate binding cleft. The binding interactions between res1 and the substrate include an aromatic pi-pi stacking interaction of the nicotinamide adenine dinucleotide phosphate (NADP) ring with the *m*-hydroquinone ring of res1. Furthermore, the substituted hydroxyls on the *m*-hydroquinone ring form H-bonds with Ser136, Asp137, Tyr159 and Arg219, Ile184. The 4-hydroxystyryl ring is H-bonded *via* its hydroxyl group to Glu193 and forms no hydrophobic interactions. Res2 binds in a deep narrow pocket within the receptor dimer interface and participates in a multitude of interactions with the monomeric A and C chains of this homopentameric complex. These include; two H-bonds *via* the *m*-hydroquinone ring to the acidic C:Glu68 and C:Asp84 residues and hydrophobic interactions with A:Val332, C:Ile81, A:Thr331, A:Arg329, C:His80. While the 4-hydroxystyryl ring makes several hydrophobic interactions like those with C:His80, A:Asn337, A:His334, C:Ala77 and does not participate in any hydrogen bonding. The binding residues for both of the resveratrol conformations belong to all three secondary structures; alpha helix, beta sheet and loop (Shafqat et al., 2013).

## Resveratrol Activating Binding Modes

### Sirt1 (PDB: 5BTR)

Sirtuins are key regulators of metabolism and activation of these enzymes by resveratrol has demonstrated therapeutic potential across several disease models (Bagul et al., 2015). Human SIRT1 encodes for sirtuin-1 with an extended N-terminal domain (NTD) and possess intrinsic mechanisms to regulate its NAD-dependent deacetylase activities (Davenport et al., 2014). Activation of Sirt1 increases lifespan by mimicking the effect of caloric restriction in several animal models (Barger et al., 2008). However, this effect has yet to be translated in primates. There is significant interest in sirtuin activation by small molecules for aging and alleviation of metabolic diseases (Howitz et al., 2003). Cell survival and enhanced metabolic activity is increased through stimulating Sirt1-dependent deacetylation of transcription several families of transcription factors, but notably PPAR-γ Coactivator 1a (PGC-1α) and Forkhead-O-Box (FOXO) (Howitz et al., 2003; Cantó and Auwerx, 2012), resulting in increased mitochondrial biogenesis, and autophagy. While controversial, it appears that resveratrol enhances the preference of Sirt1 for the bound acetylated substrate and NAD(+). Resveratrol treatment increases the rate of this deacetylation event by Sirt1 twofold, as shown in multiple *in vitro* and *in vivo* studies. Resveratrol has thus attracted attention as a prospective epigenetic therapeutic (Bagul et al., 2015).

The structure of Sirtin complex with resveratrol and a 7-amino-4-methylcoumarin (AMC)-containing peptide reveals the presence of three resveratrol molecules (res1-3), from which two mediate the interaction between the AMC peptide and the NTD of Sirt1 (Cao et al., 2015). Res1 and res1 interact with both the Sirt1 NTD and p53-AMC, while res1 contacts the Sirt1 catalytic domain (CD) and the peptide. In the res1 conformation, the *m*-hydroquinone moiety participates in H-bond interactions with Glu230 and Lys3 of p53-AMC, and hydrophobic interactions with Pro447 and Arg446. The 4-hydroxystyryl ring makes weak hydrophobic interactions with Ile 223 and Leu202 of the binding site. Res2, located at around 4–5 Å distance from res1, makes three H-bonds with Phe414, Leu215, Pro212 via its *m*-hydroquinone moiety and two with Sirt1 residues Gln222, Asn226 and one with the AMC-peptide residue, Arg1 via its 4-hydroxystyryl ring. While there are no observable hydrophobic interactions of the *m*-hydroquinone moiety with the receptor residues, two close hydrophobic interactions with the receptor residue Ile223 and the peptide residue Lys3 of the 4-hydroxystyryl ring. Res3 was located at the opposite side of the coumarin ring with respect to the locations of res1 and res2. It forms H-bonds with Asp298, Asp292 and hydrophobic interactions with Gln294, and Pro212 receptor residues via its *m*-hydroquinone ring. The 4-hydroxystyryl ring participates in a paramount H-bond with Lys444 and a hydrophobic bond with Thr209 of the receptor molecule. Interestingly, in all the three conformations the resveratrol molecules occupy a deep narrow surface cleft on the receptor molecule. The receptor binding residues strictly belong to either an alpha helix or a loop (Cao et al., 2015).

### Sirt5 (PDB: 4HDA)

Sirtuin-5 (Sirt5) is predominantly expressed in lymphoblasts and heart muscle and is a multifunctional protein that plays a key role in cellular metabolism, along with Sirt1 (Haigis et al., 2006). Resveratrol activates Sirt5 by closing the active site opening, consequently trapping the bound peptide inside the cleft which thereby increases the interaction between the peptide and Sirt5. Resveratrol binding occurs in a deep pocket, such that it is positioned next to the peptide and directly contacts its fluorophore plane. The *m*-hydroquinone ring is making a strong H-bond with Thr279 and further participates in a hydrophobic interaction with Thr278 of the receptor binding site. While the 4-hydroxystyryl ring interacts with the FdL1-peptide and makes close hydrophobic interactions with the side-chains of Arg71, Gly72 and Gln83. All major binding residues are a part of either a loop or a helix (Gertz et al., 2012).

## Resveratrol Modulatory Binding Modes

### Estrogen Receptor Alpha (PDB:4PP6)

Estrogen Receptor (ER) is activated by binding to 17β-estradiol (E2). This ligand-activated ER dimerizes and translocate in the nucleus where it indirectly impacts gene expression by modulating the activities of transcription factors such as the activator protein (AP)-1, nuclear factor-κB (NF-κB) and stimulating protein-1 (Sp-1). ER activation further exerts its effect by activating many signal transduction pathways including ERK/MAPK, p38/MAPK, PI3K/AKT, PLC/PKC (Marino et al., 2006).

Resveratrol is a pathway-selective estrogen receptor-α (ERα) ligand which aids to modulate the inflammatory response but not cell proliferation. Resveratrol binding to ERα changes the shape of the receptor in such a way that it controls which coregulatory molecules aid in regulation of transcription (Nwachukwu et al., 2014). ERα adopts a resveratrol-specific conformation due to a substantial conformational change in the receptor after resveratrol binding due to the movement and docking of multiple helices. Resveratrol binds to ERα in two different orientations (R1 and R2) which make somewhat similar interactions in each subunit of the dimer.

In R1, the resveratrol *m*-hydroquinone ring forms two H-bonds with B:Leu387 and B:Glu353 with no close hydrophobic interactions, while the 4-hydroxystyryl ring forms an H-bond with B:His524 and a hydrophobic interaction with B:Leu525. In R2, the *m*-hydroquinone ring makes close hydrophobic interactions with A:Leu525, Met421 and an H-bond with A:His524. While the 4-hydroxystyryl ring participates in several hydrophobic interactions with A:Phe404, Ala350, Leu391, Leu387 and hydrogen bonding with A:Leu387, Glu353 and Arg394.In both of the conformations, the resveratrol molecule binds into a deep pocket inside each ER monomer unit. Most residues participating in resveratrol binding belong to alpha helices, with only one in a beta sheet (Nwachukwu et al., 2014).

### Tyrosyl tRNA Synthatase (4Q93)

Tyrosyl transfer-RNA (tRNA) synthatase (TyrRS) is a 58 KD homodimeric enzyme catalyzing the aminoacylation of tRNATyr by L-tyrosine (Bedouelle, 1990). These class I synthetases play an important role in protein synthesis and signal transduction including their primary function to help in translating the genetic material into the amino-acid building blocks that make proteins (Kyriacou and Deutscher, 2008). Resveratrol inhibits the catalytic activity and redirects TyrRS to a nuclear function (Sajish and Schimmel, 2015). It does this by mimicking the natural substrate tyrosine and fits tightly into TyrRS’s tyrosine binding pocket. This binding event takes TyrRS away from its protein translation role and steers it to a function in the cell nucleus (Sajish and Schimmel, 2015).

The binding interactions between resveratrol and TyrRS predominantly involves pi-pi stacking interactions of Tyr166 and His77 via the *m*-hydroquinone ring of resveratrol. Furthermore, resveratrol also makes hydrophobic contacts with the side-chains of Ile191 and Val152 and H-bonds with Tyr166, Gln170. The 4-hydroxystyryl ring forms a pi-pi stacking interaction with His77, hydrophobic interactions with Tyr39, Leu72, Ala74 and H-bonds with Tyr39 and Asp173 residues of the TyrRS active site. Once again, the resveratrol is sitting in a deep surface cavity of the TyrRS structure. Most of the binding residues are from an alpha helix with very few in a close-by beta sheet.

## Molecular and Functional Diversity Characterizing Resveratrol’s Polypharmacology

### Target Primary Sequence and Structural Similarity

**FIGURE 2:**
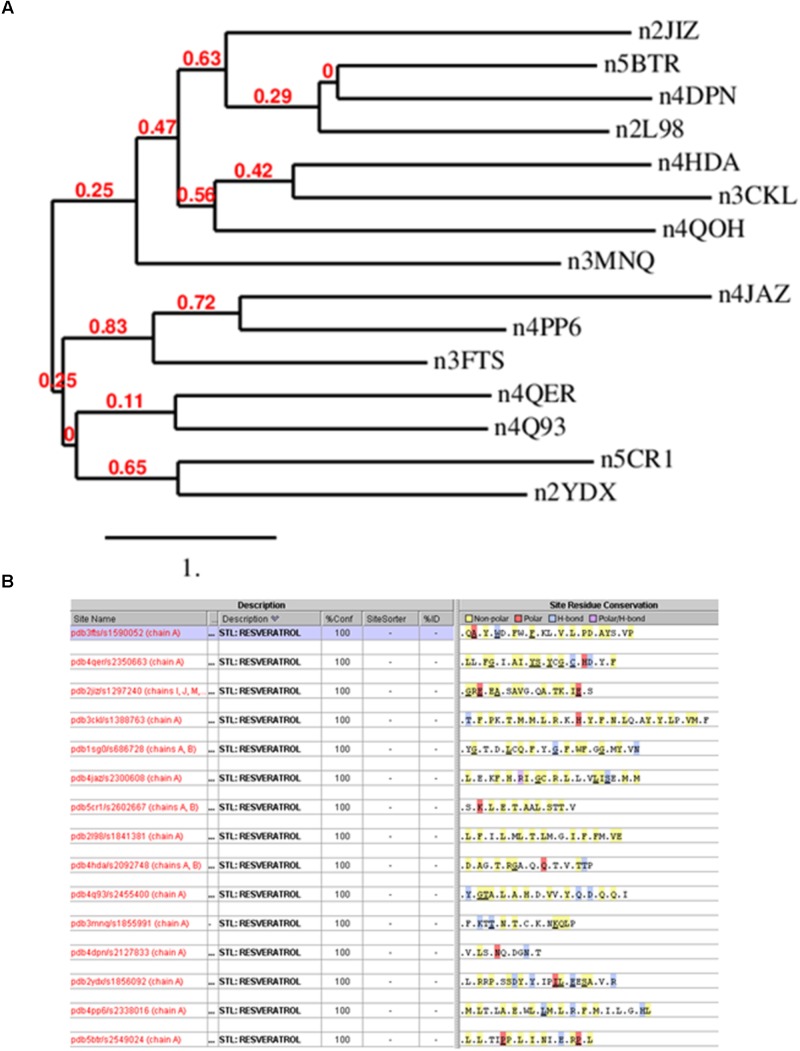
**(A)** Phylogenetic analysis of resveratrol co-crystal structures. The analysis clearly shows that there is no similarity in the resveratrol targets. Image produced by Phylogeny.fr server (http://www.phylogeny.fr/index.cgi) (Dereeper et al., 2008). **(B)** Resveratrol binding sites based on 15 resveratrol co-crystal structure chains corresponding to Table 1. Binding sites were generated by the TIP platform, Eidogen Sertanty Inc. (Hambly et al., 2006). There is no significant observed (above threshold) similarity among any of the resveratrol binding sites. Proteins shown: Leukotriene A4 hydrolase (3FTS), Phospholipase A2 (4QER), ATP synthase subunit alpha (2JIZ), Sulfotransferase family cytosolic 1B member 1 (3CKL), NRH dehydrogenase (quinone) 2 (1SG0), Peroxisome proliferator-activated receptor gamma (4JAZ), Transthyretin (5CR1), Troponin C (2I98), NAD-dependent protein deacetylase sirtuin-5 (4HDA), Tyrosine-tRNA ligase (4Q93), Myosin-2 heavy chain (3MNQ), Methionine adenosyltransferase subunit beta (2YDX), Estrogen receptor (4PP6), NAD-dependent protein deacetylase sirtuin-1(5BTR).

The simplest way to detect similarity between proteins is comparing their primary sequence. Thus, we analyzed the protein sequences of the resveratrol co-crystal structures (Supplementary Table 2) by domain sequence similarity and also by local binding site similarity. Sequence similarity is most useful when comparing functional domains or motifs, for example Pfam domains (Finn et al., 2016). The structural fold of a protein, typically also a functional domain, in most cases is largely conserved across a protein family, for example protein kinases, or nuclear receptors. Overall protein folds are typically more conserved than domain sequences. There exist protein families that share the same fold, but vary considerably in sequence similarity, for example within the GPCR transmembrane domain. Local binding site similarity is another relevant measure of relatedness that is independent of protein families defined by domain sequence or structural similarity. Simplistically, binding site similarities can be identified using just the amino acid residues subsequence of the protein that interact with a small molecule ligand or substrate. There are many methods to detect such local binding site similarity including 2D and 3D methods. Sequence identities for co-crystal structures ranged from about 0 to 29%, suggesting no significant similarity. Figure 2A shows the phylogram of all protein sequences studied. It is also clear from the diversity of the proteins binding resveratrol that they do not share a common fold. Most relevant, however, we also could not detect any similarity in the binding sites using the Target Informatics (TIP) platform from Eidogen-Sertanty, Inc. ^2^ as illustrated in Figure 2B (Hambly et al., 2006). This lack of any homology in the primary sequence, the overall fold and the properties of the local resveratrol binding residues further suggests that the targets of resveratrol are unusually diverse. These results strongly suggest the lack of a common binding pattern of resveratrol.

### Class of Targets

Typically, dual or polypharmacological inhibitors bind to the same or a similar class of targets (Reddy and Zhang, 2013). This is due to similar classes of proteins sharing some kind of structural similarity for the incoming inhibitor to bind. However, after investigating the classes of receptors modulated by resveratrol based on their structure in the PDB, it became clear that resveratrol binds to varied classes of proteins, labeling this compound as highly promiscuous. The various classes range from hydrolases (LTA4H, PLA2, F1-ATPASE, Sirt1) oxidoreductases (QR2, MAT2A), transferases (SULT1B1), Ligases (TyrRS), transcriptional regulators (PPAR-γ), Motor (Myosin-2), contractile (cTnC), and transport proteins (TTR). Since the classes of proteins are very different, one cannot determine any functional or evolutionary similarity in the resveratrol targets that could attribute to its binding to these receptors.

### Binding Residues

**FIGURE 3:**
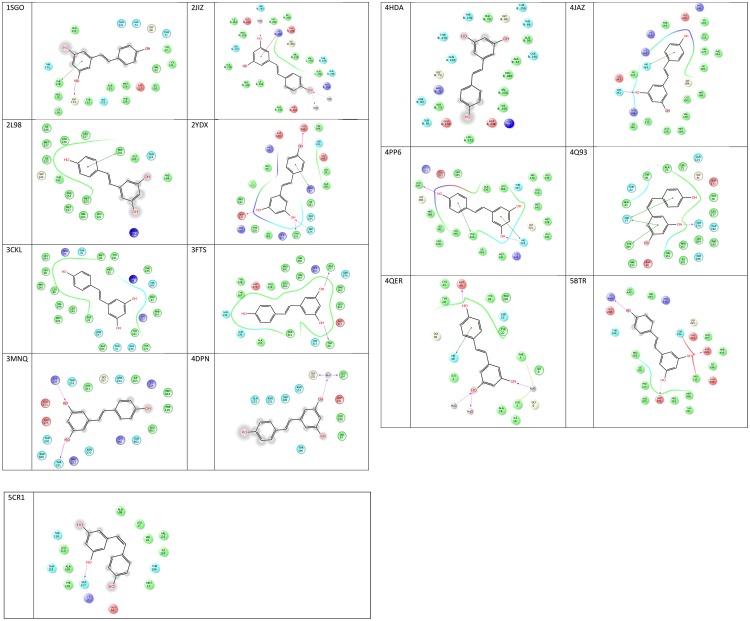
2D representations of co-crystallized resveratrol-target interactions. Image created via Ligand Interactions Viewer in the Schrodinger Maestro Suite 2017. All protein PDB structures were prepared using the Protein Preparation Wizard where likely protonation states of residues and ligand at physiological pH were generated. Green solid lines represent hydrophobic interactions, solid lines from the aromatic to other aromatic residues represent pi-stacking interactions, and solid arrows display hydrogen bonding interactions (https://www.schrodinger.com/).

Analysis of the binding residues of resveratrol in various targets indicate considerable diversity of residue type and binding interaction (Figure 3). One hypothesis suggests that resveratrol binding is mainly attributed to the hydrophobic interactions due to the presence of two benzene rings (Porat et al., 2006). However, after further investigation, such notion is not entirely true. While there are multiple pi-pi stacking and hydrophobic interactions in some targets, like SULT1B1, QR2, TyrRS, cTnC, they are very sparse in TTR and myosin-2, and almost none in the res1 conformation of MAT2B. Similarly, binding could also be attributed to the presence of substituted polar hydroxyl groups present on both rings, as in the res1 conformation of MAT2B (Shafqat et al., 2013). However, after careful observation, this also doesn’t seem to be an essential feature as contradicted by the examples of LTA4H, myosin-2 motor domain, PLA2, and Sirt1 as discussed above. In this case, there are multiple H-bonds to resveratrol hydroxyl groups *via* polar residues, but there are almost negligible H-bonds in Sirt5, TTR and even none in cTnC. Hence, in some cases the polar bonds formed between the substituted hydroxyl groups on resveratrol and receptor polar residues are extremely important in keeping the resveratrol in the active site, and in others, the two rings seem to be essential for making non-polar interactions. While still in others, there seems to be a requirement of both the polar and non-polar units of resveratrol to bind and act effectively. These binding interactions with target residues are displayed in Figure 3.

### Secondary Structure of the Binding Site Residues

**FIGURE 4:**
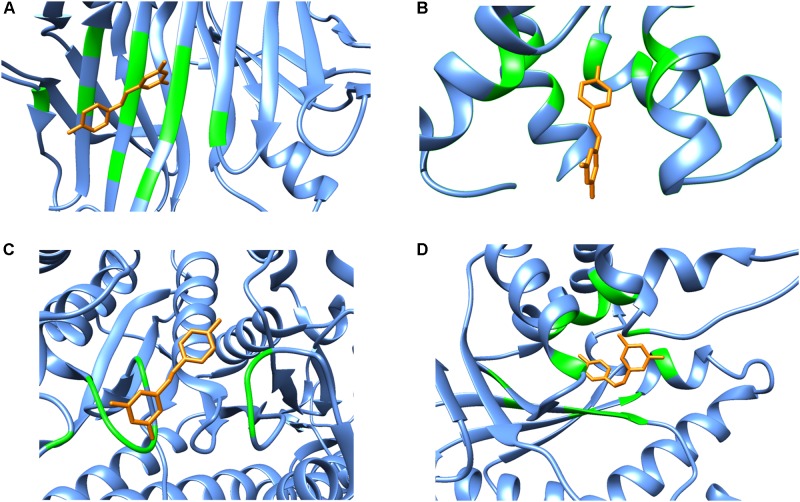
Secondary structure representations of binary complexes. Secondary structures of the resveratrol-binding residues are shown as green colored ribbons, the remaining protein residues are shown in cornflower blue ribbons and the bound resveratrol is shown as orange colored sticks. The figure shows bound resveratrol in **(A)** Transthyretin, TTR (PDB: 5CR1); **(B)** Troponin C, cTnC (PDB: 2L98); **(C)** Myosin-2 heavy chain myosin-2 motor domain (PDB: 3MNQ); and **(D)** Tyrosine-tRNA ligase, TyrRS (PDB: 4Q93) binding sites. The non-conserved mode of secondary structure elements in the receptor binding site is evident. It should be noted that the secondary structure representation is according to Chimera classification. (Image created by Chimera).

Although polypharmacology drug targets can be dissimilar in their primary domain sequence, proteins that bind to the same compound typically show similarities in residue types, the overall fold or secondary structure architecture of the binding sites (Reddy and Zhang, 2013). However, when carefully examining the secondary structure elements of resveratrol binding targets, no obvious general binding motif can be identified (Figure 4). Moreover, there is no detectable local binding site similarity using the SiteSeeker algorithm (Ecker et al., 2009) (Figure 2B). As discussed above, in most cases it is the *alpha helix and loop* residues in and around the binding cavity that form the binding brigade of the resveratrol ligand, for example PLA2, SULT1B1, QR2, Sirt5, Sirt1. However, there are few cases, where they specifically belong to a *beta sheet and loop* with none in an *alpha helix* like those in LTA4H. Interestingly, there have been some cases, where either the *alpha helix* (F1-ATPASE, cTnC) *beta sheet* (TTR) or the *loop* (myosin-2 motor domain) are involved exclusively. However, there are also instances where all three, *alpha helix, beta sheet* and the *loop* motifs are involved together (MAT2A, PPAR-γ, TyrRS). Thus, the dissimilarities in the overall fold of the binding architecture of the resveratrol receptors point to no conservation in their secondary structures.

### Binding Pocket of the Targets

**FIGURE 5:**
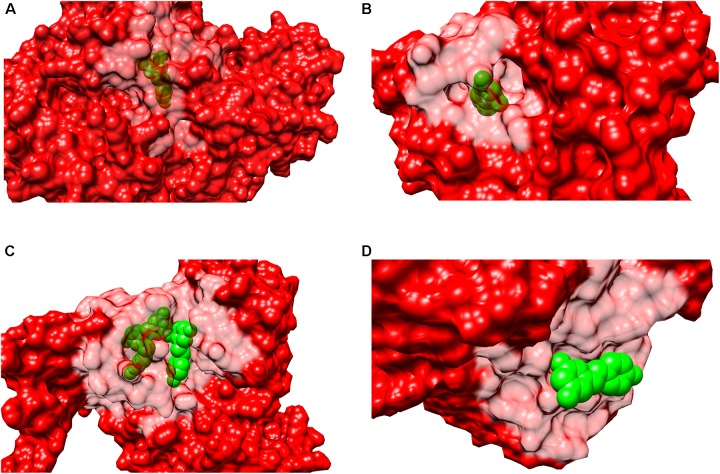
Surface representation of binary complexes. The receptors are shown as surface representation (red color) and the bound resveratrol is shown as CPK (green color). The figure above shows bound resveratrol in prototype complexes for each function: **(A)** Leukotriene A4 hydrolase, LTA4H (PDB: 3FTS), inhibitory; **(B)** Estrogen receptor, ERα (PDB:4PP6) modulatory; **(C)** NAD-dependent protein deacetylase sirtuin-1, Sirt1 (PDB: 5BTR), activatory; **(D)** Transthyretin, TTR (PDB: 5CR1), Inhibitory. The above figures highlights resveratrol’s dissimilar binding modes among different and also similar targets; including deep and shallow (surface) binding pockets.

In order to determine if resveratrol displayed a preference to a specific binding site cavity, we analyzed the 3-D space filling models for each PDB structure (Figure 5). Resveratrol is a substituted conjugated biphenyl structure, thus it could be speculated that resveratrol only binds to the deep interior cavities and pockets of the receptors. To some extent this was observed to be true in many receptors discussed previously. For example, LTA4H, F1-ATPASE, SULT1B1, QR2, PPAR-γ, Sirt5, and TyrRS. However, further analysis reveals that it is surface buried in some cases like in the case of PLA2, TTR, myosin-2 motor domain res1 conformation in MAT2A While in others, like cTnC, it binds to an open space on the receptor surface. These distinctions in binding of resveratrol to either a deep cavity inside the receptor or a shallow surface groove clearly defies the assumption of its deep interior binding.

### Mode of Action

Most of the known multi-target drugs generally inhibit or activate more than one target at the same time, but do not have the capability to activate one target and inhibit another simultaneously. However, resveratrol displays various modes of actions. We analyzed the mechanism of action of resveratrol on each target by retrieving these annotations form the ChEMBL 21 database. As discussed above, in some instances it acts as a direct inhibitor of the receptor, as seen in PPAR-γ, LTA4H, PLA2, F1-ATPASE, SULT1B1, QR2, TTR. While acting as an activator of a receptor function as in Sirt5, and Sirt1, and also as a modulator, as seen in ERα, and TyrRS. Hence, resveratrol has the ability to perform multiple functions of an inhibitor, activator or a modulator depending on the targets involved in various diseases. The details on resveratrol’s mechanism of action and pharmacology has been thoroughly discussed (Kovacic and Somanathan, 2010). All of the modes of binding described above for each target are summarized in Table 2.

**Table 2:** Structural aspects of Resveratrol binding to various targets.

Receptor protein	PDB	Binding pocket		m-hydroquinone moiety		4-hydroxystyryl moiety		Secondary structure of binding residues
				*Hydrophobic*	*H-bond*	*Hydrophobic*	*H-bond*	
LTA4H	3FTS	Deep pocket		Val367, Leu369, F314	Asp312, Phe362, Val367	Gln136, Ala137, Phe314, Pro374, Tyr378	Asp375	Loop/beta sheet
PLA2	4QER	Surface buried		Ile19	Gly6, Leu2	Phe5	Asp49, His48 Cys45	Loop or an alpha helix
F1-ATPASE	2JIZ	Deep pocket		G:Lys260, Ile263, Glu264, Ala256, Thr259 F: Val279, Ala278	F:Val279	G:Ala256, Thr259, I:Arg291	C:Glu292	Alpha helix
SULT1B1	3CKL	Deep cavity		Phe24, Phe143, Tyr170, Phe143	His109, Thr21	Leu86, Leu149, Leu244, Val248	–	Loop or an alpha helix
QR2	1SG0	Deep cavity		Phe106, Trp105, Phe178	Tyr132, Asn161	Trp105, Phe126, Tyr104	Thr71, D117	Loop or an alpha helix
PPAR-γ	4JAZ	Deep cavity		Arg288, Ile341	Ser342	Phe264, His266, Ile281	Arg280	Loop, alpha helix, beta sheet
TTR	5CR1	Shallow surface groove		Leu110	Ser117	Lys15, Ala108	–	Beta sheet
cTnC	2L98	Open pocket		Leu121, Leu100,		Leu117, leu136, Phe153, Phe156, met157, Val160	–	Alpha helix
Sirt5	4HDA	Deep pocket		Thr278, Thr69, Ala59, Gly72	Thr279	Arg71, Gln83	–	Loop, helix
TyrRS	4q93	Deep pocket		Tyr166, Ile191, His77, Val152	Tyr166, Gln170	Thr42, Gly41, Leu72, Ala74, Ala43, His77, Gln182, Tyr39	Asp173, Tyr39	Helix, beta sheet, loop
myosin-2 motor domain	3MNQ	Shallow surface groove		–	Thr231, Lys229, Thr274	Gln662, Leu663, Lys661, Asn234	–	Loop
MAT2B	2YDX	Res1: broad, shallow pocket			Ser136, Asp137, Tyr159, Arg219, Ile184		Glu193	Loop or an alpha helix, beta sheet
MAT2B ERα	2YDX 4PP6	Res2: deep narrow pocket		A:Val332, C:Ile81, A:Thr331, A:Arg329, C:His80	C:Glu68 and C:Asp84	C:His80, A:Asn337, A:His334, C:Ala77,	–	Loop or an alpha helix, beta sheet
		Deep pocket		R1	–	B:Leu387, Glu353	B:Leu525	His:B:524
ERα Sirt1	4PP6 5BTR	Deep pocket deep narrow surface cleft	R2	A:Leu525, Met421	His:A:524	A:Phe404, Ala350, Leu391, Leu387	A:Leu387, Glu353, Arg394	Alpha helix and beta sheet
			R1	A:Pro447, Arg446,	A:Glu230, D:Lys3	A:Ile 223, Leu202	–	
Sirt 1	5BTR	deep narrow surface cleft	R2	–	C:Phe414, Leu215, Pro212	F:Lys3, C:Ile223	C:Gln222, Asn226, F:Arg1	Alpha helix and loop
			R3	C:Gln294, Pro212	C:Asp298, Asp292	C:Thr209	C:Lys444	Alpha helix and loop

## Discussion

Resveratrol is only one of the numerous polyphenolic natural products found in many red grape varieties (Siemann and Creasy, 1992). In recent years, the interest in this molecule has increased exponentially following studies that demonstrated chemopreventive, cardioprotective, neuroprotective, anti-diabetic, anti-aging, and anti-inflammatory properties (Wang et al., 2002; Aziz et al., 2003; de la Lastra and Villegas, 2007; Albani et al., 2010; Bereswill et al., 2010; Campagna and Rivas, 2010; Wu et al., 2011). The myriad of beneficial activity has elicited a vast interest toward the identification of target proteins of resveratrol. Resveratrol, as a pharmacological agent, has a wide spectrum of targets, where resveratrol inhibits, activates or modulates its effects depending on the target (Hayashibara et al., 2002; Low et al., 2010; Kai et al., 2010). Herein we present an overview of the varied patterns and profiles of resveratrol target proteins and the structural aspects of resveratrol binding with its multiple targets as available in the PDB (Table 2). In order to do so, all the co-crystal structures of various targets inhibited, activated or modulated by resveratrol were extensively evaluated. We then reviewed the resveratrol polypharmacology at various levels. These include homology in primary structure and class of binding targets, mode of resveratrol binding, fold or secondary structure of the binding site, conservation in binding site residues, and mode of action (Table 2). After a detailed analysis, it appears that resveratrol displays interfamily promiscuity, which is the highest degree of promiscuity and is rarely observed in clinical compounds (Hu and Bajorath, 2013).

### Structural Modes of Binding

Sequence identity generally helps to identify common structural pattern or motifs, which may aid in understanding the binding mode of a multi-target drug or inhibitor. The protein binding site similarity is key to drug promiscuity, which also confers similar binding interactions with a common ligand. For example, Haupt et al. (2013) have demonstrated that 71% of the drugs they used in their study have at least two targets with similar binding sites. Hence similarity in the binding site is an important feature for a promiscuous drug to bind to multiple targets. However, when primary structures of the resveratrol targets were evaluated, it was observed that they do not share significant sequence identity. More importantly, based on the available resveratrol co-crystal structures, there is no significant similarity in the 3D resveratrol binding sites across the different proteins. To the best of our knowledge, this is the first review to describe the binding interactions of resveratrol on a structural level to varied scaffolds and elucidating that there is no common mode of binding to its multitude of targets or even a significant subset thereof.

After individually assessing the resveratrol-receptor interactions, it could be easily deduced that resveratrol binds varied classes of targets such as hydrolases, oxidoreductase, metal-binding proteins, transcription regulators, motor proteins, ligases contractile proteins, transferases, and transport proteins etc. Moreover, the situation is further complicated by the non-conserved mode of resveratrol binding within the pockets. This is exemplified by its binding to a deep pocket in the receptor active site as in LTA4H, F1-ATPASE, SULT1B1, QR2, PPAR-γ, Sirt5, TyrRS, while it binds to a shallow groove on PLA2, TTR, myosin-2 motor domain or even to a completely exposed cavity on the receptor surface like cTnC. Interestingly, resveratrol displays distinct binding modes even in the same receptor like as seen in MAT2A, where one molecule binds in a broad, shallow pocket while the other binds to a deep narrow pocket inside the receptor. The varied profile of receptor surface binding of resveratrol could be exemplified by Figure 5, where, it could be clearly observed that resveratrol could bind anywhere from a deep/buried to shallow/surface cavity, which might be a consequential event where local arrangement, such as conformational changes, play an integral role.

Resveratrol is composed of a conjugated phenolic ring system that attains either a planer, snugly fit, or a flat conformation inside the receptor binding site. It should be noted that the most common binding features among pre-clinical and clinical small molecules are arene ring systems substituted with hydrogen bond donors and acceptors. These phenolic rings could attribute to a series of hydrophobic interactions that serve as important forces of target binding. On this basis, this moiety could be considered a highly important and indispensable part of receptor binding by making critical non-polar hydrophobic interactions. However, closer examination of the binding site residues revealed that in some cases only hydrophobic interactions are involved between resveratrol and its targets as in TyrRS, and cTnC, but they are predominantly polar in LTA4H, myosin-2 motor domain, PLA2, Sirt1 and res1 conformation of MAT2A. Furthermore, considering that there have been instances where hydrogen bonding through the hydroxyl substituents solely constitute target binding, one might speculate the indispensability of polar hydroxyl groups for resveratrol binding to varied targets. However, as discussed above, this is not entirely true in the cases of TyrRS, and cTnC, where the binding is strictly non-polar in nature. On the other hand, there are instances where there is a mix of both polar and non-polar interactions as in TyrRS and QR2. The detailed atomic interactions of resveratrol with its binding partners have been elucidated in Figure 3, where 2-D resveratrol binding interactions with each of the targets are displayed. It is easily deduced from the figure that resveratrol does not show any specific pattern in binding site residue interactions, which could be either purely hydrophobic, polar or both.

The secondary structure pattern of resveratrol binding site residues is quite varied among its targets. While some prominently form a surrounding alpha *helix* and *loop* as in PLA2, SULT1B1, QR2, Sirt5, and Sirt1. In others, it specifically belongs to a *beta sheet* and a *loop* with no *alpha helix*, such as with LTA4H. Still others display all three secondary structures including an *alpha helix, beta sheet and a loop* jointly forming the binding residues, such as MAT2, PPAR-γ, and TyrRS. The varied profile of secondary structure elements of resveratrol targets is displayed in Figure 4. This diversity in binding may be attributed to the local conformation changes that the proteins undergo due to resveratrol binding. This is best exemplified in the case of Sirt1 activation by resveratrol discussed above, where the latter provokes a structural conformational change in the former, thereby allowing the binding of the acetylated substrate and NAD+ and ultimately enhancing the enzymatic activity. These studies point toward a dynamic mode of binding between the incoming resveratrol and the target, where conformational changes are induced as a result.

### Mode of Action

Inhibitory functions played by resveratrol are attributed to its competitive binding to the receptor for its natural substrate (LTA4H, PLA2, QR2 etc.). Resveratrol also incites activity by binding in conjugation with another activator whereby they synergistically perform receptor activation. This is exemplified by Sirt1 where two resveratrol molecules promote tighter binding between Sirt1 and the p53-AMC peptide leading to the stimulation of Sirt1 activity. Resveratrol displays its modulatory action in TyrRS by binding and hence nullifying the receptor’s catalytic activity and further directing it to a nuclear function.

Another contrasting character of resveratrol’s non-conserved mode of binding is further demonstrated by sirtuin binding. The observed mode of action is inhibitory for one class of sirtuin (Sirt2), while it plays an activator role for Sirt1 and Sirt5. Even in its activator role, there are distinct binding modes of resveratrol. Three resveratrol molecules bind to Sirt1, but only two molecules mediate the interaction between the Sirt1 N-terminal domain and the 7-amino-4-methylcoumarin (AMC)-containing peptide (p53-AMC) to stimulate Sirt1 activity. While the third resveratrol molecule further strengthens this binding by interacting with both the Sirt1 catalytic domain and the peptide. Similarly, in ERα, resveratrol binds as two different conformers, where the first conformer shows the canonical *p-*phenol of resveratrol mimicking the natural substrate, whereas in the second conformer, it is flipped. Thus, resveratrol could bind to the same protein in two or even three distinct binding modes such as in MAT2.

### Drug Development Opportunities

While there has been a multitude of impressive biological activity produced *in vitro*, and *in vivo* studies, there has yet to be an FDA approved use of resveratrol in humans. Resveratrol exerts its beneficial effects at low micromolar concentrations, owing to observed low binding constants across targets (Supplementary Table 1), coupled with overall poor ADMET (Absorption, Distribution, Metabolism, Excretion, Toxicity) properties. Because of its truly non-conserved mode of binding described at length in this review, it lacks specificity for any particular target, or interactions. Moreover, resveratrol demonstrates poor bioavailability and is rapidly excreted through metabolic hydrogenation of the aliphatic double bond, as well as glucuronidation and sulfation of the hydroxyl moieties (Wang and Sang, 2018).

However, considering the demonstrated therapeutic potential of resveratrol, there has been significant interest in synthesizing more potent derivatives with increased binding and improved ADMET. For example, aza-modified compounds developed by Fujita et al resulted in an increase in potency (Fujita et al., 2012), and a series of bridged stilbene derivatives demonstrated submicromolar inhibition of both COX-1 and COX-2 (Handler et al., 2007). In yet another study, resveratrol stilbene derivatives with modifications at the 4’ position of the β-ring were synthesized with higher potency for Sirt1 activation and lifespan extension in *Saccharomyces cerevisiae* (Howitz et al., 2003). Bioavailability was increased with analogue 3,4,5,4′-tetrahydroxystilbene, although elimination is also likely to increase (Lu et al., 2001). Sirtris, acquired by GlaxoSmithKlein in 2008, was a company dedicated to creating sirtuin activators, and patented a proprietary resveratrol formulation to improve bioavailability by utilizing microsphere particles, but no improvement in activity was obtained from either efforts (Schmidt, 2010). Yet, another strategy to improve the pharmacological benefits of resveratrol is through a prodrug approach. One promising design was the usage of amino acid carbamates to partially, or fully modify the hydroxyl groups linked via an N-monosubstituted carbamate ester bond to derivatives of glycerol or galactose (Biasutto et al., 2014). Thus, conferring higher water solubility, improving bioavailability, and offering initial protection from quick metabolic degradation, and elimination.

There has even been significant interest by natural product chemists to isolate, characterize and synthesize a more potent resveratrol-like natural product that is a part of the same, or a stress-response, biosynthetic pathway. These compounds are known as secondary metabolites and are not essential for the survival of the organism, but are produced in small amounts due to an external trigger. While this approach may not necessarily account for positive changes to ADMET, they may display superior, and more targeted biological activity. The overarching obstacle for this approach is obtaining these complex molecules in appreciable amounts, as extraction would be nearly impossible due to the negligible amounts produced by the organism throughout its lifetime. Several research groups have successfully synthesized oligomeric resveratrol natural products in quantities needed for biological testing. Snyder et al. (2011) accessed Caraphenol A in gram scale quantities, along with efficiently synthesizing several other compounds with promising *in vitro* activity, such as Pallidol, Ampelopsin F, Vaticanol A, and Hopeahainol A. More recently, Keylor et al. (2016) developed a controlled radical methodology to efficiently access resveratrol tetramers such as Nepalensinol B, and Vateriaphenol C. However, it is yet to be demonstrated if these natural products will offer any improved activity *in vivo*, and in primate models.

While there have been no real marked advances in translating the intriguing bioactivity of resveratrol into an efficacious drug via a typical medicinal chemistry approach. Global systems-wide analysis such as genome-wide transcriptional responses of the effects of resveratrol offer an alternative approach. Recently, researchers have displayed interest in investigating resveratrol’s activity at a systems level. Fang et al. (2017) predicted possible other targets of interest for the anti-cancer effects of resveratrol based on statistical analysis of publicly available databases of deposited bioactivity (such as ChEMBL, Binding DB, and TCGA) combined with by machine learning. Crossland et al. (2017) observed that while resveratrol did reverse transcription of genes that are the most perturbed due to aging in patient derived muscle cells and tissue, it did not reverse the disease (aging) gene signature when they observed the effect at a systems level. Interestingly, the authors state that based on their findings, SIRT1 and AMPK may not be a regulator of healthy aging, further explaining the lack of translational activity for resveratrol.

The Library of Integrated Cellular Signatures (LINCS) is an NIH common fund project established in 2013 that has the underlying goal to understand the effect of perturbagens at cellular level for disease and health states (Keenan et al., 2018). Resveratrol is one of many perturbagens tested in the L1000 high-throughput reduced representation genome-wide transcriptional profiling assay developed by The Broad Institute enabling rapid transcriptional profiling of large compound libraries across many cell lines (Subramanian et al., 2017). Analysis of this data is being carried out in hopes of better understanding on a holistic level how resveratrol, and other perturbagens, modulate disease. For example, the transcriptional impact across many cell lines perturbations with similar profiles (e.g., connectivity) can be explored via the Clue platform at the Broad Institute^3^. All LINCS datasets are available via the LINCS Data Portal (Koleti et al., 2018). Analysis of this data is also of interest to another NIH common fund project, Illuminating the Druggable Genome (IDG), which seeks to discover drug targets that are currently underexplored or even unknown. Through the analysis of “big” genome-wide profiling datasets and potential novel targets, it may be possible to better understand the mechanisms, including novel pathways and possibly novel protein targets that underlie resveratrol’s beneficial health effects. All of these would improve our understanding of Nature’s micromolar love-affair with resveratrol and guide the way to the development of novel therapeutics that overcome promiscuity and finally harness its polypharmacological potential and associated beneficial health effects.

## Conclusion

Resveratrol, (trans-3,4′,5-trihydroxystilbene) is a polyphenolic phytoalexin present in many plants. It has gained much attention since the 1980’s, but translation of resveratrol’s intriguing bioactivity from the bench to the clinic has yet to be demonstrated. There are multiple reasons for the failure to translate activity in primates, including the interfamily promiscuity, coupled with poor drug-like physicochemical and ADMET properties. However, because of the broad therapeutic profile of this phytochemical, there are multiple ongoing efforts to exploit its pharmacology and fully tap its pharmaceutical potential, despite a generally low observed activity against its targets.

In the current review, we have covered almost all of the co-crystal structures of resveratrol involved in inhibition, activation and modulation of the target proteins associated with various diseases. The analyses of resveratrol with its targets revealed that they share no significant similarity in domain sequence, structural fold or even the local 3D binding site. Thorough analysis of various resveratrol-receptor complexes reveals that the compound does not have any preferred mode of binding. This provides a clue to its heterogeneous nature of binding where it has no special preference to a hydrophobic groove or a charged surface. Investigation of the co-crystal structures of resveratrol-protein complexes at the atomic level also revealed its non-conserved mode of binding. Analyses of binding patterns reveal that the compound has no preference to acidic, basic or neutral amino acids and could potentially bind to any or all of the categories. Similarly, the secondary structures of resveratrol’s binding partners have little in common, with binding site architecture ranging from primarily *alpha helix, beta sheet*, or merely *loops* or in some cases a combination of all three. Even the mode of action of resveratrol is diverse in different targets, where it displays inhibitory, activatory and modulatory activity.

After careful analysis of the various aspects of target binding, one cannot form a consensus of resveratrol’s binding characteristics, and it should be regarded as a highly promiscuous compound. Although, one feature that is clear from its mode of action is its positive impact on diseases. Through inhibition, activation or modulation resveratrol displays varied therapeutic potential. A different approach of analyzing resveratrol’s impact on disease from a systems biology level may yield new insights for utilizing resveratrol-like compounds as polypharmacological medicine.

All LINCS data and curated bioactivity data of resveratrol are available from the LINCS Data Portal at http://lincsportal.ccs.miami.edu/SmallMolecules/view/LSM-42917.

## Author Contributions

US and TK are both first co-author and both contributed equally to writing this manuscript. SP, DL, RS, MB, and SS all contributed to the manuscript and the analysis and presentation of the various resources presented.

## Conflict of Interest Statement

The authors declare that the research was conducted in the absence of any commercial or financial relationships that could be construed as a potential conflict of interest.

## Footnotes

1 https://www.schrodinger.com/

2 http://www.eidogen.com/tip.php

3 https://clue.io/
