# Supplementary material for: Polypharmacology or Promiscuity? Structural Interactions of Resveratrol With Its Bandwagon of Targets
Source: Front Pharmacol. 2018 Oct 24;9:1201. doi: 10.3389/fphar.2018.01201 (PMC6207623; doi:10.3389/fphar.2018.01201)
Supplement: Supplementary file 1 [file Table_1.DOCX]

**Supporting Information**

**Polypharmacology or Promiscuity? Structural Interactions of Resveratrol with its Bandwagon of Targets**

**Uzma Saqib^1‡^, Tanya T. Kelley^2‡^, Panguluri Siva Kumar^3^, Dongfang Liu^4^, Rajkumar Savai^5^, Mirza S. Baig^6*^, Stephan C. Schürer^2,7*^**

^1^Discipline of Chemistry, Indian Institute of Technology-Indore, MP, India

^2^Department of Molecular and Cellular Pharmacology, University of Miami Miller School of Medicine, Miami, FL, USA

^3^Department of Pharmaceutical Science, University of South Florida, Tampa, FL, USA

^4^Center for Inflammation & Epigenetics, Houston Methodist Research Institute, Houston, TX, USA

^5^Max Planck Institute for Heart and Lung Research, Department of Lung Development and Remodeling, Member of the German Center for Lung Research (DZL), Bad Nauheim, Germany

^6*^Department of Bioscience and Bioengineering, Indian Institute of Technology-Indore, MP, India

^7*^Center for Computational Science, University of Miami, Coral Gables, FL, USA

**‡** First Co-Authors

* Corresponding Authors,

Mirza S. Baig ([msb@iiti.ac.in](mailto:msb@iiti.ac.in)), Stephan C. Schürer ([sschurer@miami.edu](mailto:sschurer@miami.edu)**)**

**Supplemental Table 1:** Resveratrol bioactivity data (-Log10 transformed) by functional, biochemical and ADMET assays.


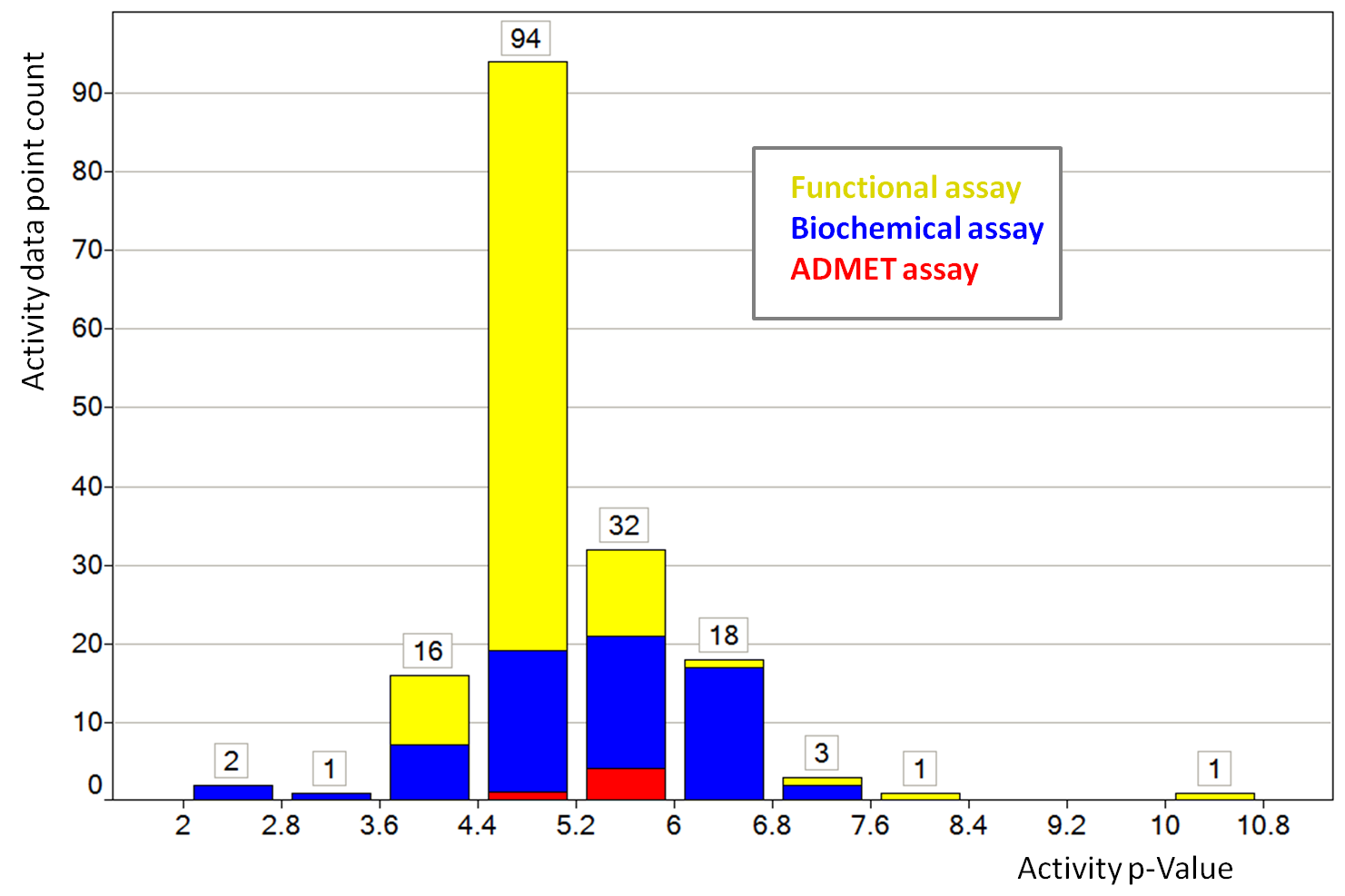


These values were obtained via querying the ChEMBL 21 Database, followed by filtering for *Homo sapiens*, exact end point values, and confidence scores equal or greater than 5. Data points were aggregated as an arithmetic mean for the unique targets, and reported endpoints.

**Supplemental Table 2**: Percent Identity Matrix for the PDB Structures Mentioned in This Review.

Created by Clustal2.1

|  | | 1SG0 | 4QER | 3FTS | 3CKL | 4JAZ | 4Q93 | 3MNQ | 2L98 | 4PP6 | 4HDA | 5BTR | 2YDX | 2JIZ | 4DPN | 5CR1 |
| --- | --- | --- | --- | --- | --- | --- | --- | --- | --- | --- | --- | --- | --- | --- | --- | --- |
| 1 | 1SG0 | 100 | 12.33 | 18.18 | 8.33 | 14.29 | 15.48 | 22.3 | 17.86 | 11.29 | 6.2 | 11.33 | 16.26 | 12.68 | 10.26 | 11.11 |
| 2 | 4QER | 12.33 | 100 | 10.81 | 5.13 | 12.5 | 11.54 | 15.15 | 9.09 | 12.5 | 18.46 | 8.33 | 12.5 | 6.02 | 5.88 | 10 |
| 3 | 3FTS | 18.18 | 10.81 | 100 | 15.67 | 13.88 | 15.57 | 20.13 | 18.92 | 7.73 | 9.94 | 13.36 | 10.71 | 18.09 | 14.74 | 6.76 |
| 4 | 3CKL | 8.33 | 5.13 | 15.67 | 100 | 15.87 | 14.89 | 19.37 | 12.5 | 12.62 | 4.5 | 8.38 | 11.04 | 9.57 | 9.86 | 6.12 |
| 5 | 4JAZ | 14.29 | 12.5 | 13.88 | 15.87 | 100 | 15.25 | 20.4 | 12.82 | 20.44 | 4.17 | 12.24 | 10.07 | 7.14 | 12.5 | 8.57 |
| 6 | 4Q93 | 15.48 | 11.54 | 15.57 | 14.89 | 15.25 | 100 | 20.96 | 12.96 | 13.89 | 3.95 | 10.45 | 7.52 | 9.8 | 10.64 | 7.69 |
| 7 | 3MNQ | 22.3 | 15.15 | 20.13 | 19.37 | 20.4 | 20.96 | 100 | 18.46 | 18.75 | 7.32 | 16.99 | 11.52 | 12.35 | 8.65 | 9.09 |
| 8 | 2L98 | 17.86 | 9.09 | 18.92 | 12.5 | 12.82 | 12.96 | 18.46 | 100 | 20.83 | 10 | 14 | 13.33 | 8.16 | 12.5 | 0 |
| 9 | 4PP6 | 11.29 | 12.5 | 7.73 | 12.62 | 20.44 | 13.89 | 18.75 | 20.83 | 100 | 10.08 | 17.32 | 8.97 | 10.81 | 8.7 | 8.96 |
| 10 | 4HDA | 6.2 | 18.46 | 9.94 | 4.5 | 4.17 | 3.95 | 7.32 | 10 | 10.08 | 100 | 29.37 | 15.15 | 14.72 | 13.04 | 10.34 |
| 11 | 5BTR | 11.33 | 8.33 | 13.36 | 8.38 | 12.24 | 10.45 | 16.99 | 14 | 17.32 | 29.37 | 100 | 14.48 | 14.22 | 14.52 | 13.13 |
| 12 | 2YDX | 16.26 | 12.5 | 10.71 | 11.04 | 10.07 | 7.52 | 11.52 | 13.33 | 8.97 | 15.15 | 14.48 | 100 | 22.86 | 18.18 | 11.43 |
| 13 | 2JIZ | 12.68 | 6.02 | 18.09 | 9.57 | 7.14 | 9.8 | 12.35 | 8.16 | 10.81 | 14.72 | 14.22 | 22.86 | 100 | 23.53 | 18.81 |
| 14 | 4DPN | 10.26 | 5.88 | 14.74 | 9.86 | 12.5 | 10.64 | 8.65 | 12.5 | 8.7 | 13.04 | 14.52 | 18.18 | 23.53 | 100 | 18.81 |
| 15 | 5CR1 | 11.11 | 10 | 6.76 | 6.12 | 8.57 | 7.69 | 9.09 | 0 | 8.96 | 10.34 | 13.13 | 11.43 | 18.81 | 18.81 | 100 |
